# Decoding host-microbe interactions with engineered human organoids

**DOI:** 10.1038/s44318-025-00387-3

**Published:** 2025-02-21

**Authors:** Lucas A Meirelles, Alexandre Persat

**Affiliations:** 1 Global Health Institute, School of Life Sciences, École Polytechnique Fédérale de Lausanne (EPFL), Lausanne, Switzerland; 2 Institute of Bioengineering, School of Life Sciences, École Polytechnique Fédérale de Lausanne (EPFL), Lausanne, Switzerland

**Keywords:** Microbiology, Virology & Host Pathogen Interaction

## Abstract

This commentary of the *Sparks of Science* series from the Catalysts program discusses the insights into pathogen behavior provided by human organoid studies in combination with high throughput approaches.

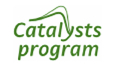

## Teaser

As the antimicrobial resistance crisis becomes a reality, we are only now realizing we lack a fundamental understanding of the mechanisms by which major pathogens infect their human host. To address this, we must employ novel methodologies that allow us to investigate microbial behavior in human infection-like contexts. Here, we present a framework for studying host-microbe interactions by integrating human-derived organoids with high-resolution live imaging and high-throughput sequencing technologies. This interdisciplinary and multiscale strategy has the potential to resolve host-microbe interactions in great detail under conditions replicating those encountered in vivo. Thus, infection studies based on organoid models could open new avenues for fundamental and translational research with therapeutic potential.

Microbes inhabit a wide variety of environments, each with unique chemical and physical signatures. To explore how bacteria colonize and adapt to these diverse niches, microbiologists often adjust laboratory culture conditions to mimic some of their specific features. However, in vitro laboratory conditions only rarely replicate the physicochemical complexity of natural microbial habitats exhaustively, limiting our understanding of microbial behavior “in context”. The development of new culture methods that recapitulate some of these missing features, therefore, has the potential to reveal new facets of microbial physiology.

The discrepancy between laboratory conditions and natural environments is especially true for human bacterial pathogens. Animal models currently constitute the gold standard for validating in vitro mechanistic studies, as they can approach the complexity of an entire organism. However, they face major limitations (Alonso-Roman et al, 2024; Pound and Ritskes-Hoitinga, 2018; Clark, 2018). First, physiological and histological differences compared to humans often limit the relevance of animal infections. Second, navigating experimental and ethical constraints remains challenging. For example, our limited ability to perform in situ imaging in mammalian models severely limits our ability to monitor infection progression in real time. Imaging explants can partially address this problem, but these tissues are short-lived and cannot be maintained (Alonso-Roman et al, 2024).

To overcome the experimental limitations of test tubes and animal models, infection biologists often turn to cell cultures. Immortalized cell lines are often used in the investigation of bacterial pathogenesis to identify virulence factors (Alonso-Roman et al, 2024). They are easy to maintain and genetically manipulate. However, cancer cell lines display a drastically altered physiology compared to native healthy cells. In addition, since these cells cannot differentiate, they fail to replicate the diverse histological signatures of healthy tissues. Nevertheless, they can be integrated into organ-on-chip (OoC) systems—microfluidic devices that emulate the physical features of the host (Alonso-Roman et al, 2024; Mishra et al, 2023; Grassart et al, 2019; Benam et al, 2016). Traditional OoCs, however, often use abiotic material substrates as tissue interfaces, which lack the biological structure found in vivo. Further development of advanced in vitro infection models that integrate proper cell types and replicate tissue function could bridge laboratory studies, animal experiments, and clinical investigations.

When cultured with appropriate growth factors and within an extracellular matrix (ECM) scaffold, stem cells grow and differentiate into multicellular structures called organoids, whose histological signatures closely resemble those of human tissues (Sato et al, 2009; Spence et al, 2011). For example, intestinal stem cells can self-organize into intestinal organoids composed of enterocytes, goblet cells, enteroendocrine cells, stem cells, and Paneth cells in the shape of crypts and villi (Serra et al, 2019). Similarly, lung organoids also exhibit all the cell types found in the airway epithelium, including basal cells, ciliated cells, goblet cells, and club cells (Sachs et al, 2019). Organoids can be grown from multipotent primary cells derived from patients or from induced pluripotent stem cells (iPSCs). Organoids are tractable and can also be genetically engineered, particularly when generated from iPSCs, to produce human organoid mutant lines (Hofer and Lutolf, 2021). While access to human primary and stem cells had been a limiting factor, the democratization of iPSCs, biobanks, and commercial kits have recently improved the access of human organoids to non-specialized labs.

Human-derived organoids thus offer a relevant and realistic environment for microbial colonization, closely resembling the conditions they would encounter during infection of a human host. Therefore, organoids could advance the investigation of infections in virtually any tissue. We envision that they will become especially relevant to mucosal surfaces of the lungs and intestine, two sites where host–microbe interactions have been hard to study mechanistically. Here, we discuss applications of human-derived engineered organoids in infection biology and how these could couple with powerful omics technologies.

## Tissue-engineered human organoids for investigations of microbial physiology

Although early organoid models have been instrumental in developmental biology, their cystic morphology—with a luminal space facing inward and embedded in hydrogel matrices—limits access to the lumen. Organoids grown in this configuration cannot clear their luminal debris of dead cells and secreted material, which affects their viability (Hofer and Lutolf, 2021). To study bacterial interactions with their mucosal surface, an experimenter must, therefore, microinject microbes into the lumen, one organoid at a time. This procedure affects epithelial integrity, is labor-intensive, and has limited throughput. To enable direct access to their mucosal surface and avoid microinjection, organoids can switch polarity so that their apical surface faces outward (Co et al, 2019). So-called “apical-out” intestinal organoids have been employed to study patterns of cellular invasion by *Salmonella* Typhimurium and *Listeria monocytogenes* (Co et al, 2019). Although powerful for studying intracellular intestinal pathogens, this method is not suitable for primarily extracellular pathogens or for other organs, like the lung, where an air-liquid interface is crucial in pathogen infectivity patterns.

Setting up “open-face” organoids can alleviate some of these limitations. In this configuration, organoid monolayers grow on a synthetic porous membrane with their apical side exposed to microbes, providing direct access to the surface of the tissue (Puschhof et al, 2021). Commercial Transwells allow integration in well-plate formats, making this configuration scalable and attractive for applications requiring medium to high throughput. Commercial synthetic membranes, however, scatter light heavily, complicating live microscopy. Furthermore, the inserts must remain submerged in medium, which obstructs high-resolution microscope objective access. Finally, Transwell-grown tissues are closed systems contained in a well or dish, which impacts microbial colonization dynamics. Increasing the morphological accuracy and imaging compatibility of these tissues, particularly for live infections, could enhance their tractability as human infection models.

New approaches wherein a scaffold guides stem cell differentiation can help control organoid morphology and organization, including apical accessibility (Jiménez-Torres et al, 2016; Nikolaev et al, 2020; Hofer and Lutolf, 2021). Manufacturing procedures enable precise control of organoid shape, for example, to produce tubular morphologies. Furthermore, control of lumen positioning allows for designs that improve compatibility with in situ high-resolution live microscopy. To emulate a realistic lung environment and monitor infections live, we engineered tube-shaped lung organoids called AirGels. We obtained a tube morphology by first patterning a cavity within an ECM scaffold, all contained in a 3D-printed device (Rossy et al, 2023). Human primary bronchial epithelial cells subsequently expand at the surface of the ECM tube, then differentiate at the air-liquid interface to produce an airway epithelial tissue with the histological signature of a human lung, including mucus production and active cilia beating in a tubular morphology compatible with high-resolution live microscopy. Live imaging at high spatiotemporal resolution in AirGels was essential to reveal that the pathogen *Pseudomonas aeruginosa* contracts mucus with type IV pili to rapidly form biofilms while colonizing the mucosal airway (Rossy et al, 2023).

The development of models compatible with high-resolution imaging and improved accessibility is an area of active research. For instance, an approach to build patterned gastrointestinal organoid monolayers with easy bilateral access (e.g., basal and apical) was recently developed (Hofer et al, 2024). The system, called Transgels, combines the characteristics of epithelial monolayers grown in Transwell devices with the flexibility of patterning hydrogel scaffolds. The authors developed epithelial monolayers from distinct mouse stomach, cecum, colon, and small intestine tissues, and imaged gastrointestinal infections by the parasite *Trichuris muris* (Hofer et al, 2024). This system could be easily adapted to support human gastrointestinal cells and study their interactions with pathogenic or commensal species.

Overall, organoids and engineered organoids replicate tissue histological and physiological signatures to unprecedented levels. By recapitulating the cell types found in vivo, they mimic the molecular responses and biophysical characteristics of the original tissue within a controlled environment. In addition, these models can be augmented, for example, by incorporating functional immune cells (Noel et al, 2017; Recaldin et al, 2024). Thus, a bottom-up approach in engineering organoids for microbiological applications holds great potential for their democratization as infection models. As a result, they could provide a human-centric understanding of the molecular mechanisms driving host-pathogen interactions.

## Combining high-throughput technologies with organoids: a new perspective on infections

Given the emulating power and tractability of tissue-engineered organoids, investigating their colonization at high spatial, physical, and biological resolutions could help identify previously unresolved mechanisms of interaction between host and microbes. High-throughput genomic and proteomic technologies can broadly assess bacterial physiology (Westermann et al, 2017; Starr et al, 2018). In these experiments, it is necessary to initially calibrate the infection inoculum and dynamics, which can be done with imaging. By observing the progression of infection dynamics, it is possible to visualize how distinct populations grow in space and time, defining the infection stages to be characterized. One can then isolate or sort the population of interest—such as bacteria attached to mucus or cells that invaded the tissue—and perform guided sequencing at selected time points. Readouts will depend on the specific questions. For example, infections of engineered tissues followed by RNA-seq or proteomics may reveal bacterial physiological adaptations in infection contexts that are highly controlled, unlike animal models. Additionally, implementing genome-wide fitness screens, such as transposon-insertion sequencing (Tn-seq) or CRISPRi screens (Cain et al, 2020; Todor et al, 2021; Russell et al, 2024), within organoids allows for comparing fitness and gene essentiality during infection, tracking pathogen evolution, identifying adaptation strategies, and even uncovering novel drug targets.

We used Tn-seq in human airway mucosal tissues derived from primary human bronchial epithelial cells to study *P. aeruginosa* adaptation strategies to the mucosal surface at the onset of infection, revealing that genes involved in biofilm lifestyle and metabolism were crucial for bacterial fitness under these conditions (Meirelles et al, 2024). More importantly, the experiment showed that *P. aeruginosa* physiology in mucus differed significantly from that in test tube cultures. Consequently, mutations affecting fitness more closely replicated clinical observations from *P. aeruginosa* evolution in patients, supporting the potential of organoid models to replicate aspects of human physiology and advance discovery.

In a similar spirit, a functional genomics Tn-seq screen for *Mycobacterium abscessus* growing at an air-liquid interface infection model derived from a bronchial epithelial immortalized cell line revealed that biotin biosynthesis was required for colonization (Sullivan et al, 2023). The authors demonstrated *M. abscessus* has an increased need for intracellular biotin during infection. Consistent with this requirement, inhibition of biotin biosynthesis with pharmacological drugs prevented colonization (Sullivan et al, 2023).

Subsequent imaging can then test hypotheses generated from high-throughput screens. Engineered organoid models are particularly suited for microscopy applications. Thus, specific hits from functional genomics, transcriptomics, or proteomics experiments can be further investigated using individual mutant strains or reporters. For example, we used live imaging in AirGels to track the colonization mechanisms of *P. aeruginosa* mutants identified by Tn-seq (Meirelles et al, 2024). Detailed live imaging in engineered organoids allows dynamic monitoring of spatial heterogeneity—an essential feature for understanding mechanisms driving colonization, virulence, or treatment susceptibility. This new way of studying infections allowed us to observe and quantify host cell death dynamics as well as population features associated with high antibiotic tolerance.

**Figure 1:**
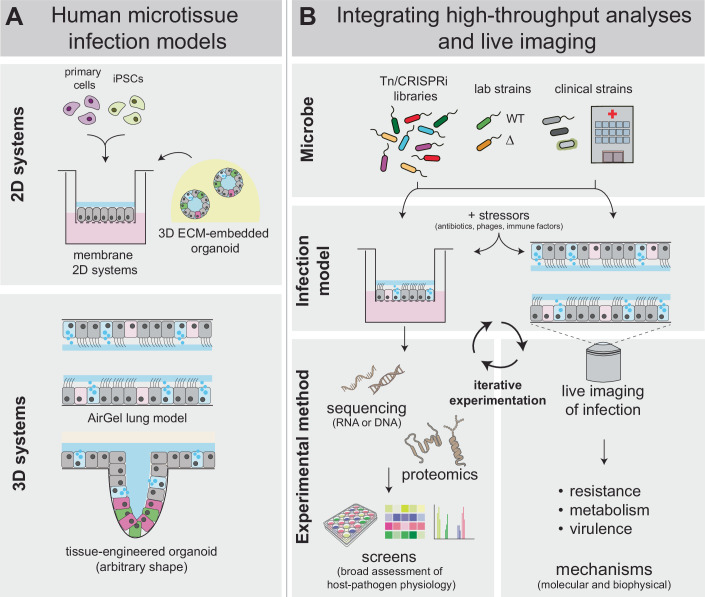
Decoding bacterial physiology and host–microbe interactions by integrating high-throughput analyses and live imaging in engineered human tissues. Recent advances in organoids and tissue engineering allow the development of new infection models that accurately recapitulate multiple aspects of the infection environment in human hosts (Rossy et al, 2023; Nikolaev et al, 2020) (**A**). This is particularly relevant for mucosal surfaces, such as those in the gut and lung. These models can be used to study the physiology of the pathogen or commensal of interest (i.e., their growth and survival under stressors, such as antibiotics, phages, or immune factors), as well as how these bacteria interact with host cells (**B**). Iterative experimentation between more simplistic 2D models used for screens, and more mechanistic experiments with live imaging in engineered tissues has the potential to reveal novel physiological aspects of how these microbes behave in in vivo-like environments. Some of the illustrations shown in (**B**) were modified from the NIH BIOART catalog (https://bioart.niaid.nih.gov). Used illustrations are # 4, 73, 129, 453, and 468.

The combination of Tn-seq and imaging in engineered organoids is adaptable to a wide range of pathogens and commensals present in mucosal tissues, allowing for a broad assessment of fitness determinants in these environments. A key aspect of this approach is the intricate connection between high-throughput sequencing in 2D organoid cultures and high-resolution experiments in engineered 3D tissues (Fig. 1A,B). Balancing throughput and imaging resolution is often necessary, and iteratively experimenting with the two configurations is most powerful for mechanistic investigations.

How can we reconnect organoid experiments with human infections? Genome-wide fitness screens can help explain the fitness costs and benefits of mutations observed in clinical isolates and link findings to clinical manifestations of infections. For instance, *P. aeruginosa* is known to adapt to the lung environment and antibiotic treatment (Rossi et al, 2021; Marvig et al, 2015). As an illustration, combining Tn-seq and live imaging enabled the identification of trade-offs associated with mutations driving biofilm formation found in *P. aeruginosa* during human lung infections (Meirelles et al, 2024). Other pathogens and infection contexts could benefit from such an approach. When built from patient-derived cells, these models could serve as powerful tools for personalized medicine. For example, linking patient-specific host features to bacterial adaptation using tissues and clinical strains from the same patient may reveal individual vulnerabilities that could be targeted for therapeutic interventions.

In summary, integrating engineered human tissue models with high-throughput methods and live imaging creates a powerful pipeline for advancing our understanding of microbial physiology and host-pathogen interactions during infections (Fig. 1). In Box 1, we propose a few outstanding general questions in microbiology that could be resolved with the contributions of human microtissues. To best address each question, the infection model must be carefully tailored. In the future, we envision that increased throughput will enable the implementation of engineered organoids to large-scale screen with ordered transposon libraries or drug libraries (Sollier et al, 2024).

Box 1. Open questions addressable with engineered organoids **Bacteria-focused questions:**

- What core genes are essential for a pathogen to grow in any specific tissue microenvironments?
- How do physical features of the mucosal surface impact pathogenicity?
- Which specific cell types and receptors do pathogens target during infection?
- How do microbes adapt their metabolism to the nutrient composition found in the tissue environment?

**Host-focused questions:**

- How do epithelial tissues and specific cell types respond to infections?
- What specific signaling pathways are activated in the host during infections?
- How do innate immune cells shape the mucosal environment and affect infection dynamics?

## Supplementary information

Peer Review File
