# Supplementary material for: Decoding host-microbe interactions with engineered human organoids
Source: EMBO J. 2025 Feb 21;44(6):1569–73. doi: 10.1038/s44318-025-00387-3 (PMC11914615; doi:10.1038/s44318-025-00387-3)
Supplement: Supplementary file 1 — Peer Review File [file 44318_2025_387_MOESM1_ESM.pdf]

THE EMBO JOURNAL

Decoding host-microbe interactions with engineered human organoids

Lucas Meirelles and Alexandre Persat

Corresponding author: Alexandre Persat (alexandre.persat@epfl.ch)

Review Timeline:

Submission Date:	28th Jan 25
Editorial Decision:	29th Jan 25
Revision Received:	5th Feb 25
Accepted:	6th Feb 25

Editor: Ieva Gailite

Transaction Report:

This article was editorially reviewed.
